# Cigarette smoke extract upregulates heme oxygenase-1 via PKC/NADPH oxidase/ROS/PDGFR/PI3K/Akt pathway in mouse brain endothelial cells

**DOI:** 10.1186/1742-2094-8-104

**Published:** 2011-08-24

**Authors:** Ruey-Horng Shih, Shin-Ei Cheng, Li-Der Hsiao, Yu Ru Kou, Chuen-Mao Yang

**Affiliations:** 1 Department of Pharmacology, Chang Gung University, Tao-Yuan, Taiwan; 2 Department of Physiology, National Yang Ming University, Taipei, Taiwan

## Abstract

**Background:**

In the brain, the inducible form of heme oxygenase (HO-1) has been recently demonstrated to exacerbate early brain injury produced by intracerebral hemorrhagic stroke which incident rate has been correlated with cigarette smoking previously. Interestingly, cigarette smoke (CS) or chemicals present in CS have been shown to induce HO-1 expression in various cell types, including cerebral endothelial cells. However, the mechanisms underlying CS modulating HO-1 protein expression are not completely understood in the brain vessels.

**Objective:**

The aim of the present study was to investigate the mechanisms underlying CS modulating HO-1 protein expression in cerebral endothelial cells.

**Methods:**

Cultured cerebral endothelial cells (bEnd.3) were used to investigate whether a particulate phase of cigarette smoke extract (PPCSE) regulates HO-1 expression and to investigate the molecular mechanisms involved in HO-1 expression in bEnd.3 cells.

**Results:**

We demonstrated that PPCSE (30 μg/ml) significantly induced HO-1 protein expression and its enzymatic activity in bEnd.3 cells determined by western blotting and bilirubin formation, respectively. PPCSE-induced HO-1 expression was mediated through phosphatidylcholine phospholipase C (PC-PLC), PKCδ, and PI3K/Akt which were observed by pretreatment with their respective pharmacological inhibitors or transfection with dominant negative mutants of PKCδ and Akt. ROS scavenger (N-acetyl-L-cysteine, NAC) blocked the PPCSE-induced ROS generation and HO-1 expression. Pretreatment with selective inhibitors of PKCδ (rottlerin) and NADPH oxidase [diphenyleneiodonium chloride (DPI) and apocynin (APO)] attenuated the PPCSE-induced NADPH oxidase activity, ROS generation, and HO-1 expression. In addition, we found that PPCSE induced PI3K/Akt activation via NADPH oxidase/ROS-dependent PDGFR phosphorylation.

**Conclusions:**

Taken together, these results suggested that PPCSE-induced HO-1 expression is mediated by a PC-PLC/PKCδ/NADPH oxidase-dependent PDGFR/PI3K/Akt pathway in bEnd.3 cells.

## Background

Cigarette smoke (CS) is a complex aerosol that can be separated into gas and particulate phases. Particulate phase of CS extract (PPCSE), such as lipophilic components, could pass the lipid bilayer of respiratory membranes [1] into blood stream. Therefore, the damage of CS not only limits to lung tissue but also vascular system. CS is a known risk factor not only for peripheral cardiovascular inflammation-related diseases [2,3] but also for cerebrovascular diseases, such as stroke [4].

In the brain, the inducible form of heme oxygenase (HO) plays as a rate-limiting enzyme in the process of oxidative degradation of pro-oxidant heme into potent antioxidants [5]. HO-1 has been recently demonstrated to exacerbate early brain injury produced by intracerebral hemorrhagic stroke [6]. Interestingly, CS or chemicals present in CS have been shown to induce HO-1 expression in various cell types [7-10] including cerebral endothelial cells [11]. Therefore, PPCSE-induced HO-1 expression in cerebral vessels might be an important factor for brain injury in hemorrhagic stroke which incident rate has been correlated with cigarette smoking previously [4].

High levels of reactive components and radicals present in CS [12,13] trigger cytotoxic effects with increasing dosages in various cell types [14,15]. Different facets of CS-induced cytotoxic stress have been extensively studied in peripheral endothelial cells. Previous studies have reported that CS-induced HO-1 expression was mediated through activation of PKC [16], p42/p44 MAPK [17], JNK1/2 [10], PI3K/Akt [18], transcription factor Nrf2 [7], CdRE [19], and Bach1 [8] in various cell types. Recently, we also found the involvement of NADPH oxidase/ROS/PDGFR pathway in PPCSE-induced HO-1 expression in mouse cultured brain endothelial cells (bEnd.3) [11]. However, the molecular mechanisms underlying PPCSE-induced HO-1 expression were not completely understood in cerebral vessels.

In the present study, we used PPCSE to investigate the molecular and cellular mechanisms of PPCSE-induced HO-1 expression in bEnd.3. Our results demonstrated that PPCSE-induced HO-1 expression was mediated through a PKCδ/NADPH oxidase/ROS/PDGFR/PI3K/Akt cascade.

## Methods

### Materials

Anti-HO-1 antibody was purchased from Santa Cruz (Santa Cruz, CA). Anti-phospho-PDGFR and anti-phospho-Akt antibodies were purchased from Cell Signaling (Danver, MA). Anti-PKC-δ and anti-Akt antibodies were purchased from Santa Cruz (Santa Cruz, CA). AG1296, diphenyleneiodonium chloride (DPI), LY294002, wortmannin (Wort), GF109203X, rottlerin (Rott), Gö6976, U73122 and D609 were purchased from Biomol (Plymouth Meeting, PA). Apocynin (APO) was purchased from ChromaDex (Santa Ana, CA). CM-H_2_DCFDA was from Molecular Probes (Eugene, OR). N-acetyl-L-cysteine (NAC) and other chemicals were purchased from Sigma (St. Louis, MO).

### Preparation of PPCSE

PPCSE was prepared with modification of previously described method [20,21]. In brief, 10 cigarettes (Long Life™, Taiwan Tobacco and Liquor Production, Taipei, Taiwan; 0.9 mg nicotine/cigarette) were combusted and the smoke was sucked through a standard glass-fiber enabled Cambridge filter with a constant flow of 0.3 liters/min with an air compressor and this filter has been reported to trap > 99% of the smoke particulate matter [20]. The smoke filter was quickly dried with a hot plate and the raised weight in each filter was defined as the amount of PPCSE. On average, each cigarette smoke generated ~39.5 mg of PPCSE using this method. The PPCSE was dissolved by soaking the filter in DMSO for 30 min at room temperature. The solution containing PPCSE was centrifuged and the supernatant was collected and filtered using a 0.22-μm filter column (Millipore, Bedford, MA). The PPCSE stock solution (20 mg/ml of PPCSE containing 0.36 mg/ml of nicotine) was divided into microtubes (each 15 μl) and stored immediately at -80°C. Before each experiment, the frozen PPCSE stock solution was defrosted and further diluted to the desired concentration with cell medium. The quality of the PPCSE solution was assessed based on the absorbance at 302 nm, which is the specific absorption spectrum of peroxynitrite. Therefore, the concentration of DMSO in the testing solution was always < 1% to prevent possible cytotoxicity or other effects.

### Cell culture

Mouse brain endothelial cells (bEnd.3) were purchased from Bioresource Collection and Research Centre (BCRC 60515; Hsinchu, Taiwan) and grew in DMEM/F-12 containing 10% FBS and antibiotics (100 U/ml penicillin G, 100 μg/ml streptomycin and 250 ng/ml fungizone) at 37°C in a humidified 5% CO_2 _atmosphere. When the cultures grown to confluence (about 4 days), cells were released with 0.05% (w/v) trypsin/0.53 mM EDTA for 5 min at 37°C. The cell suspension was diluted with DMEM/F-12 containing 10% FBS to a concentration of 2 × 10^5 ^cells/ml. The cell suspension was plated onto 6-well culture plates (2 ml/well) and 10-cm culture dishes (10 ml/dish) for the measurement of protein and RNA expression, respectively. Culture medium was changed in every 24 h of the 3 consecutive days. Experiments were performed with cells from passages 5 to 13.

### Enzymatic assay for HO-1 activity

HO activity was measured based on the levels of bilirubin formation using a microsomal fraction of cells as a source [22]. Briefly, bEnd.3 cells were homogenized and sonicated before centrifuging at 15000 × *g *for 40 min at 4°C. The supernatant (500 μg of protein) was mixed with NADH-containing buffer (1 mM NADH, 2 mM glucose 6-phosphate, 2 U glucose-1-phosphate dehydrogenase, 600 μg of biliverdin reductase, 1 mM potassium phosphate buffer, and 25 μM hemin) for 60 min at 37°C in the dark. The reaction without NADH was served as a blank. The reactions were stopped by placing the mixture on ice and subsequently scanned with a spectrophotometer. The amount of bilirubin was determined by the difference in optical density units between 470 and 550 nm.

### Determination of ROS production

The intracellular H_2_O_2 _levels were determined by measuring fluorescence of DCFH-DA. For the purpose of these experiments, cells were washed with warm HBSS and incubated in HBSS containing 10 μM DCFH-DA at 37°C for 45 min. Subsequently, HBSS containing DCFH-DA was removed and replaced with a fresh medium. Cells were then treated with PPCSE with/without pretreatment of a ROS scavenger or inhibitors of NADPH oxidase. Cells were washed twice with PBS and detached with trypsin/EDTA, and the fluorescence intensity of the cells was analyzed using a FACScan flow cytometer (BD Biosciences, San Jose, CA) at 495 nm excitation and 529 nm emission for DCF.

### Determination of NADPH oxidase activity by chemiluminescence assay

The NADPH oxidase activity in intact cells was assayed by lucigenin chemiluminescence assay, as previously described [23] with modifications. After incubation, cells were gently scraped and centrifuged at 400 × g for 10 min at 4°C. The cell pellet was resuspended with 35 μl/per well of ice-cold RPMI 1640 medium, and the cell suspension was kept on ice. To a final 200 μl volume of pre-warmed (37°C) RPMI 1640 medium containing NADPH (1 μM) or lucigenin (20 μM), 5 μl of cell suspension (0.2 × 10^5 ^cells) was added to initiate the reaction followed by immediate measurement of chemiluminescence in an Appliskan luminometer (Thermo^®^) in an out-of-coincidence mode. Appropriate blanks and controls were established, and chemiluminescence was recorded. Neither NADPH nor NADH enhanced the background chemiluminescence of lucigenin alone (30-40 counts per min). Chemiluminescence was continuously measured for 12 min, and the activity of NADPH oxidase was expressed as counts per million cells.

### PC-PLC activity assay

PC-PLC activity assay was determined as described previously [24]. In brief, we prepared the enzyme and used L-α-phosphatidylcholine (Sigma) as the substrate of phosphatidylcholine-specific phospholipase C (PC-PLC). The optical density was measured at wavelength of 660 nm. The enzyme activity was expressed as nmol per min per mg of protein (nmol/min/mg protein).

### PKC Kinase Activity Assay

Protein kinase C activity was performed according to instructions of manufacturer (Enzo Life Science, #ADI-EKS-420A), which is based on a solid phase enzyme linked immuno-absorbent assay (ELISA) that utilizes a specific synthetic peptide as a substrate for subtypes of PKCs, including δ, α, β, η, and ζ, and a polyclonal antibody that recognizes the phosphorylated form of the substrate. The optical density was measured at wavelength of 450 nm. The enzyme activity was expressed as a relative activity.

### Western blot analysis

After incubation, cells were washed with ice-cold PBS, homogenization buffer A (20 mM Tris-HCl, pH 8.0, 10 mM EGTA, 2 mM EDTA, 2 mM dithiothreitol, 1 mM phenylmethylsulfonyl fluoride, 25 μg/ml aprotinin, 10 μg/ml leupeptin) was added and the cells were scraped into a 1.5-ml tube with a rubber policeman. The suspension was sonicated for 10 s at output 4 with a sonicator (Misonix, Farmingdale, NY) and centrifuged at 8000 rpm for 15 min at 4°C. The supernatant was collected as the cytosol with cellular membrane fractions and the pellet as the nuclear fraction. The supernatant was then centrifuged at 14,000 rpm for 60 min at 4°C to separate cytosol fraction and cellular membrane fraction. The pellet was resuspended in 300 μl of homogenization buffer B (1% Triton X-100 in buffer A) and sonicated for 10 s. The suspension was centrifuged at 14,000 rpm for 10 min at 4°C. The supernatant was collected as a nuclear lysate fraction. On the other hand, after the incubation, cells were washed, scraped, collected, and centrifuged at 45000 × *g *at 4°C for 1 h to yield the whole cell extract. The denatured samples were subjected to 10% SDS-PAGE and the protein fractions were transferred to nitrocellulose membrane. Membranes were incubated with desired primary antibodies for 24 h at 4°C, and then incubated with horseradish peroxidase-conjugated anti-goat antibody for 1 h at room temperature. The immunoreactive bands were developed with ECL reagents. GAPDH served as internal controls for all the experiments.

### Transient transfection with dominant negative plasmids

Cells were cultured in 12-well culture plates. The plasmid encoding dominant negative mutant of PKC-δ or Akt was prepared by using QIAGEN plasmid DNA preparation kits and was formulated with Metafectene transfection reagent according to the manufacturer's instruction (Biontex Lab., Planegg/Martinsried, Germany). The amount of tansfection plasmid was kept constant (2 μg of dominant negative mutant of PKCδ or Akt for each well). The cells were shifted to serum-free DMEM/F-12 for 24 h before exposure to PPCSE.

### Akt Activity Assay

Akt activity Akt was determined by using an Akt activity assay kit (BioVision, #K45-40). The cell lysates were prepared according to instructions of manufacturer. Akt immunoprecipitation was conducted by using an Akt antibody and protein A sepharose beads, washed by kinase assay buffer, and then incubated with GSK-3α protein/ATP mixture. The phosphorylation of GSK-3α protein was analyzed by 12% SDS-PAGE and blotted using an anti-phospho-GSK-3α antibody. The activated Akt exerted as a positive control was generated by incubation with 20% FBS for 30 min.

### Statistical analysis

Data were calculated using GraphPad Prism Program (GraphPad, San Diego, CA). Quantitative data were analyzed using one-way analysis of variance (ANOVA) followed by Tukey's honestly significant difference tests between individual groups. Data were expressed as mean ± SEM. A value of *P *< 0.05 was considered to be significant.

## Results

### PPCSE induces HO-1 expression and enzymatic activity

**Figure 1:**
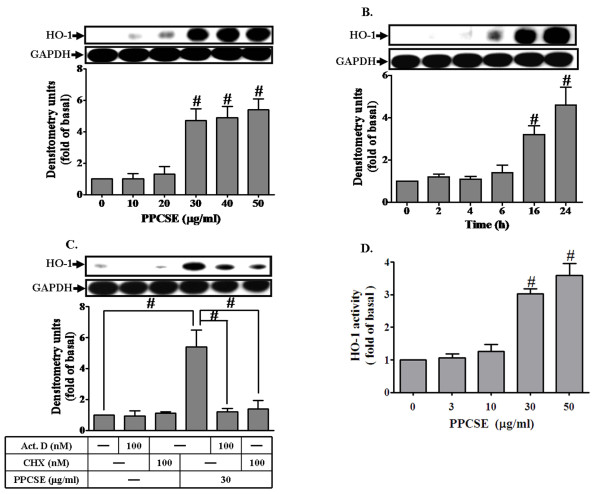
**PPCSE induces HO-1 protein and activity in bEnd.3 cells**. **A**: Cells were treated with various concentrations of PPCSE for 24 h. **B**: Cells were treated with 30 μg/ml PPCSE for the indicated time intervals. **C**: Cells were pretreated with actinomycin D (Act.D) or cycloheximide (CHX) for 1 h, and then incubated with PPCSE for 24 h. **A, B, C**: The expression of HO-1 was determined by Western blot analysis. **D**: Cells were treated with various concentrations of PPCSE for 24 h, and then the activity of HO-1 was determined. Data are expressed as the mean ± SEM of three individual experiments. **A, B, D**: ^#^*P *< 0.05 as compared with the cells exposed to vehicle alone. **C**: ^#^*P *< 0.05 as compared with the cells exposed to PPCSE alone.

To determine the effect of PPCSE on HO-1 expression, cells were incubated with various concentrations of PPCSE for the indicated time intervals. As shown in Figures 1A and 1B, PPCSE induced HO-1 expression in a concentration- and time-dependent manner, with a maximal response at a concentration of 30 μg/ml for 24 h. PPCSE also induced HO-1 mRNA accumulation in a time-dependent manner with a maximal response within 6 h (data not shown). To further determine if PPCSE-induced HO-1 expression required either transcriptional or translational process, a transcriptional level inhibitor (actinomycin D, Act.D) and a translational level inhibitor (cycloheximide, CHX) were used for these purposes. As shown in Figure 1C, pretreatment with either Act.D or CHX attenuated PPCSE-induced HO-1 expression in a concentration-dependent manner. These findings demonstrated that the induction of HO-1 by PPCSE depends on *de novo *protein synthesis. In addition, we found that PPCSE concentration-dependently increased HO-1 enzyme activity in bEnd.3 cells (Figure 1D).

### Involvement of NADPH oxidase in PPCSE-induced HO-1 expression

**Figure 2:**
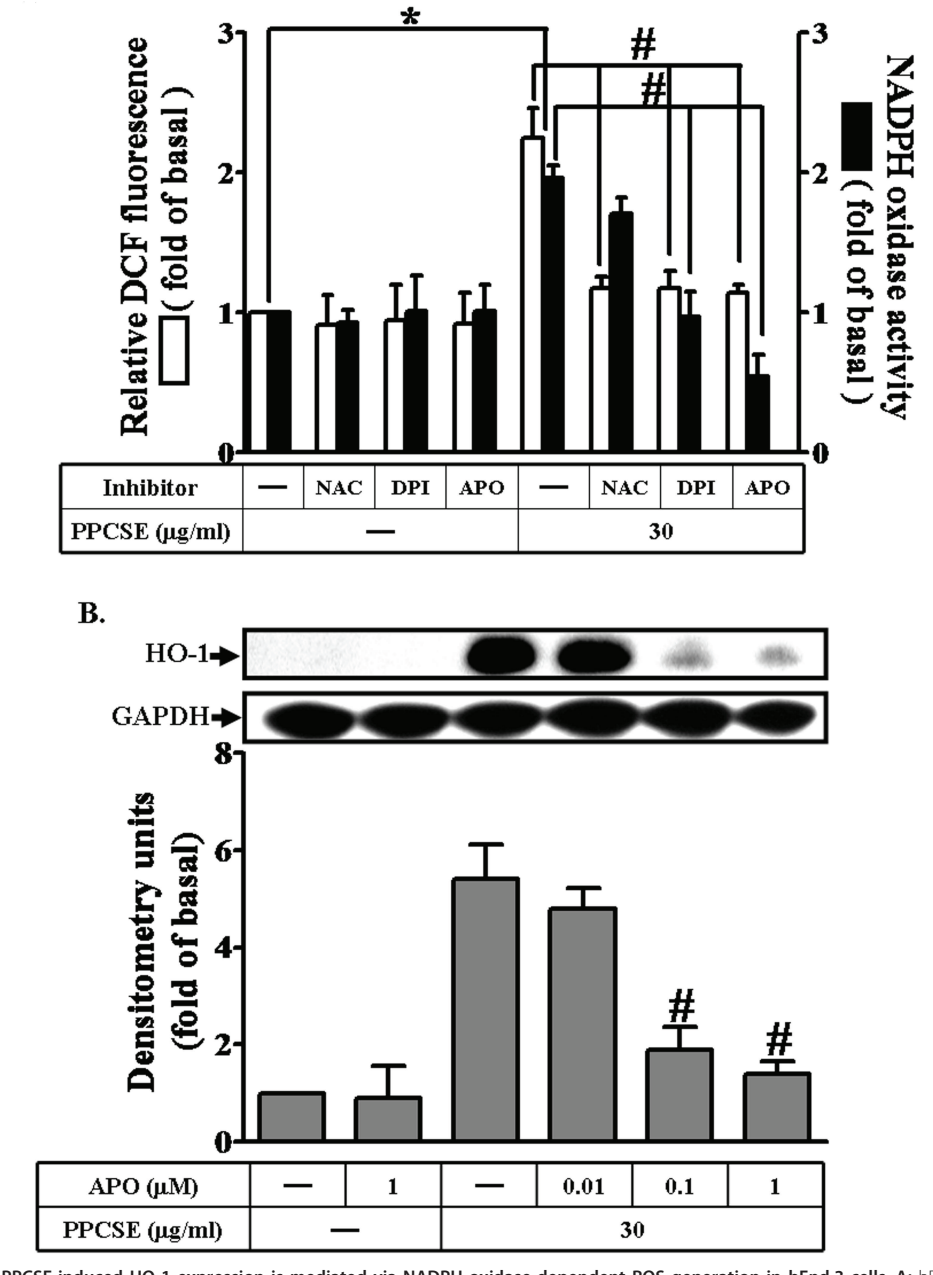
**PPCSE-induced HO-1 expression is mediated via NADPH oxidase-dependent ROS generation in bEnd.3 cells**. **A: **bEnd.3 cells were labeled with DCF-DA (10 μM), and then incubated with 30 μg/ml PPCSE for 30 min with/without pretreatment of NAC (3 mM), DPI (1 μM) or APO (1 μM) for 1 h. The fluorescence intensity (relative DCF fluorescence) was measured at 495 nm excitation and 529 nm emission using a FACScan flow cytometer. The NADPH oxidase activity was measured as described in the Methods. **B: **Cells were pretreated with APO for 1 h, and then incubated with PPCSE for 24 h. The expression of HO-1 was determined by Western blot. Data are summarized and expressed as the mean ± SEM of three individual experiments. *P < 0.05 as compared with the cells exposed to vehicle alone. ^#^*P *< 0.05 as compared with the cells exposed to PPCSE alone.

PPCSE has been shown to induce the expression of inflammatory proteins associated with various inflammatory responses [25,26]. NADPH oxidase is an important enzymatic source for the production of ROS under various pathological conditions. The ingredients of PPCSE also induce endogenous ROS generation via a NADPH oxidase pathway in endothelial cells [27]. To determine if PPCSE also induced intracellular ROS generation in bEnd.3 cells, DCF-DA was used as a fluorescence indicator to measure the intracellular ROS levels. As shown in Figures 2A, PPCSE markedly increased ROS production and NADPH oxidase activity within 30 min, which were attenuated by pretreatment with NAC (a scavenger of ROS) or the inhibitors of NADPH oxidase (DPI and APO). In addition, PPCSE-induced HO-1 protein expression was inhibited by pretreatment with APO (Figure 2B). These results suggested that PPCSE-induced HO-1 expression was mediated through NADPH oxidase-dependent ROS generation in bEnd.3 cells.

### PPCSE-induced HO-1 expression is mediated by a PC-PLC/PKCδ/NADPH oxidase cascade

**Figure 3:**
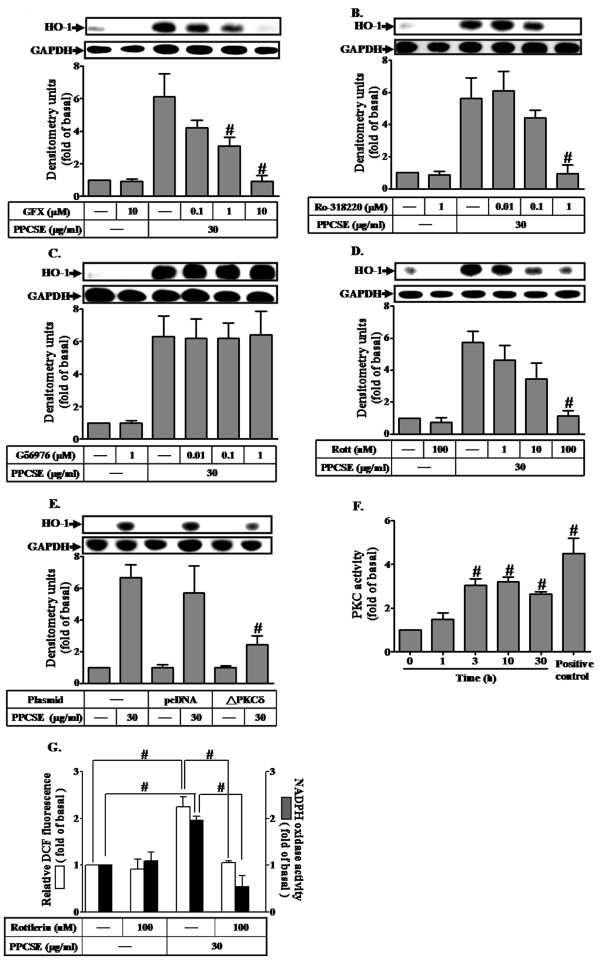
**PPCSE-induced HO-1 expression is mediated by PKCδ in bEnd.3 cells**. **A-D: **Cells were pretreated with non-selective inhibitors of PKC, GF109203X (GFX) and Ro318220 (Ro), and Gö6976 (Gö) or selective inhibitor of PKCδ, rottlerin (Rott) for 1 h, and then incubated with PPCSE for 24 h. The expression of HO-1 was determined by Western blot. **E: **Cells were transfected with a dominant negative mutant of PKCδ (**Δ**PKCδ), and then incubated with PPCSE for 24 h. The expression of HO-1 was determined by Western blot. **F: C**ells were treated with 30 μg/ml PPCSE for the indicated time intervals, and then the activity of PKC was determined as described in the Methods. **G: **Cells were pretreated with rottlerin (Rott) for 1 h, and then incubated with PPCSE for 1 h. The ROS generation and NADPH oxidase activity were measured. Data are expressed as the mean ± SEM of three independent experiments performed in triplicate. **A-F: **^#^*P *< 0.05 as compared with the cells exposed to PPCSE alone. **G: **^#^*P *< 0.05 as compared within groups.

To investigate the role of PKC in PPCSE-induced HO-1 expression, cells were pretreated with the non-specific inhibitors of PKC (GF109203X and Ro318220), inhibitor of calcium-dependent PKC (Gö6976) or inhibitor of PKCδ (rottlerin) prior to the incubation with PPCSE for 24 h. As shown in Figures 3A-D, pretreatment with GF109203X (GFX), Ro318220 (Ro), or rottlerin (Rott), but not Gö6976 (Gö), markedly inhibited PPCSE-induced HO-1 expression, suggesting that Ca^2+^-independent PKCs such as PKCδ would have predominant role in PPCSE-induced HO-1 expression. To further ensure the role of PKCδ in the expression of HO-1 induced by PPCSE, cells were transfected with a dominant negative mutant of PKCδ. As shown in Figure 3E, transfection with a dominant negative mutant of PKCδ significantly attenuated PPCSE-induced HO-1 expression. The activity of PKC was increased within 3-10 min in response to PPCSE, determined by an ELISA kit (Figure 3F). Pretreatment with rottlerin also decreased NADPH oxidase activity and ROS generation (Figure 3G).

**Figure 4:**
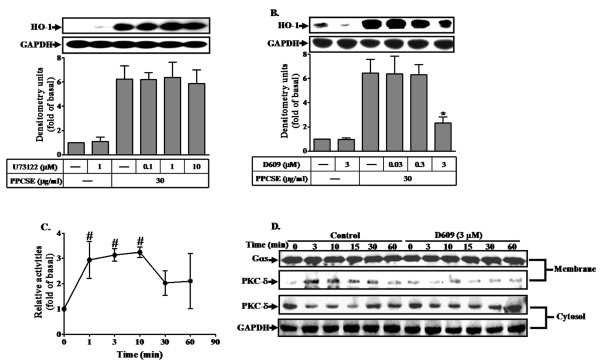
**PPCSE-induced HO-1 expression is mediated via a PC-PLC/PKCδ signaling in bEnd.3 cells**. A, B: Cells were pretreated with the inhibitor of PI-PLC (U73122) or PC-PLC (D609) for 1 h, and then incubated with PPCSE for 24 h. The expression of HO-1 was determined by Western blot. C: Cells were treated with 30 μg/ml PPCSE for the indicated time intervals, and then the activity of Ca^2+^-independent PC-PLC was determined as described in the Methods. The basal level of Ca^2+^-independent PC-PLC was 27.4 ± 3.44 nmol/min/mg protein. **D**: Cells were pretreated with D609 for 1 h, and then incubated with PPCSE for the indicated time intervals. The cytosolic and membrane fractions were prepared and subjected to 12% SDS-PAGE to determine the translocation of PKCδ using an anti-PKCδ antibody. Data are summarized and expressed as the mean ± SEM of three individual experiments. *P < 0.05 as compared with the cells exposed to PPCSE alone. ^#^*P *< 0.05 as compared with basal level.

PKC can be activated by DAG which was cleavage from PIP_2 _catalyzed by phospholipase C (PLC) enzymes [28]. To determine whether the expression of HO-1 induced by PPCSE was mediated via phosphatidylcholine PLC (PC-PLC) or phosphatidylinositide PLC (PI-PLC), cells were pretreated with the inhibitor of PI-PLC (U73122) or PC-PLC (D609), and then incubated with PPCSE for 24 h. As shown in Figures 4A and 4B, pretreatment with D609, but not with U73122, inhibited PPCSE-induced HO-1 expression, suggesting that PPCSE-induced HO-1 expression is mediated through Ca^2+^-independent PC-PLC. This note was further supported by a PC-PLC activation assay, as shown in Figure 4C, PPCSE increased PC-PLC enzymatic activity within 1-3 min. These results suggested that the expression of HO-1 induced by PPCSE was mediated via Ca^2+^-independent PC-PLC in bEnd.3 cells. In addition, PKCδ was translocated to cell membrane in a time-dependent manner with a maximal response within 3-10 min under PPCSE stimulation, which was attenuated by D609 (Figure 4D). These results indicated that PPCSE-induced HO-1 expression was mediated through a PC-PLC/PKCδ-dependent signaling pathway.

### Akt plays an important role in PPCSE-induced HO-1 expression

**Figure 5:**
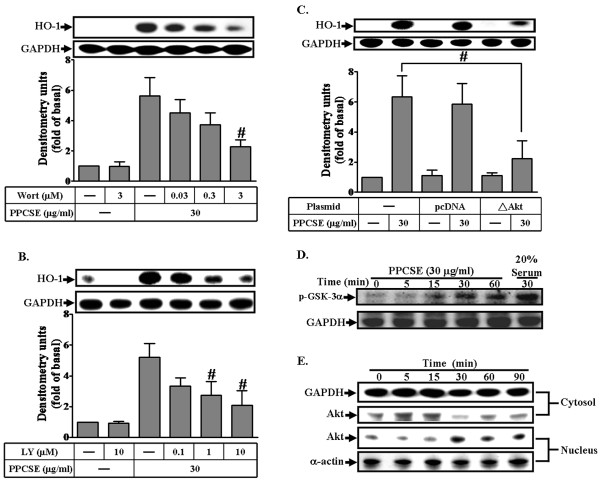
**Akt mediated HO-1 induction by PPCSE**. **A, B**: Cells were pretreated with wortmannin (Wort) or LY294002 (LY) for 1 h, and then incubated with PPCSE for 24 h. The expression of HO-1 was determined by Western blot. **C**: Cells were transfected with a dominant negative mutant of Akt (ΔAkt), and then incubated with PPCSE for 24 h. The expression of HO-1 was determined by Western blot. **D**: Akt kinase activity was determined as described in the Methods. Cells were treated with 30 μg/ml PPCSE for the indicated time intervals. Phosphorylation of GSK-3α was used to determine the activity of Akt stimulated by PPCSE and analyzed by western blot. **E**: Time dependence of PPCSE-stimulated Akt translocation into nucleus. Cells were treated with 30 μg/ml PPCSE for the indicated time intervals. The cytosolic and nuclear fractions were analyzed by Western blot using an anti-Akt, anti-GAPDH (as a cytosol control), or anti-α-actin (as a nuclear control) antibody. **A-C**: Data are expressed as the mean ± SEM of three individual experiments. ^#^*P *< 0.05 as compared with the cells exposed to PPCSE alone. **D, E**: Similar results were obtained from three independent experiments.

We have recently shown that PDGFR plays a role in PPCSE-induced HO-1 expression in bEnd.3 cells [11]. In order to elucidate whether PI3K/Akt, which is a well known downstream component of PDGFR, involved in PPCSE-induced HO-1 expression, the inhibitors of PI3K, wortmannin and LY294002, were used. As shown in Figures 5A and 5B, PPCSE-induced HO-1 expression was attenuated by pretreatment with either wortmannin (Wort) or LY294002 (LY) in a concentration-dependent manner. To ensure the involvement of PI3K/Akt in PPCSE-induced HO-1 expression, an Akt dominant negative mutant was used. As shown in Figure 5C, transfection of cells with a dominant negative mutant of Akt attenuated PPCSE-induced HO-1 expression in bEnd.3 cells. We also detected the Akt kinase activity stimulated by PPCSE. As shown in Figure 5D, the Akt activity, which was indicated by phosphorylated-GSK-3α, was increased in a time-dependent manner with a maximal response within 30 min. We further determined whether PPCSE-induced responses were mediated through Akt which was translocated into nucleus. Our results showed that PPCSE stimulated translocation of Akt in a time-dependent manner (Figure 5E). These data indicated that PPCSE-induced HO-1 expression was mediated through a PI3K/Akt-dependent pathway in bEnd.3 cells.

### Involvement of NADPH oxidase/ROS in sequential activation of PDGFR/PI3K/Akt induced by PPCSE

**Figure 6:**
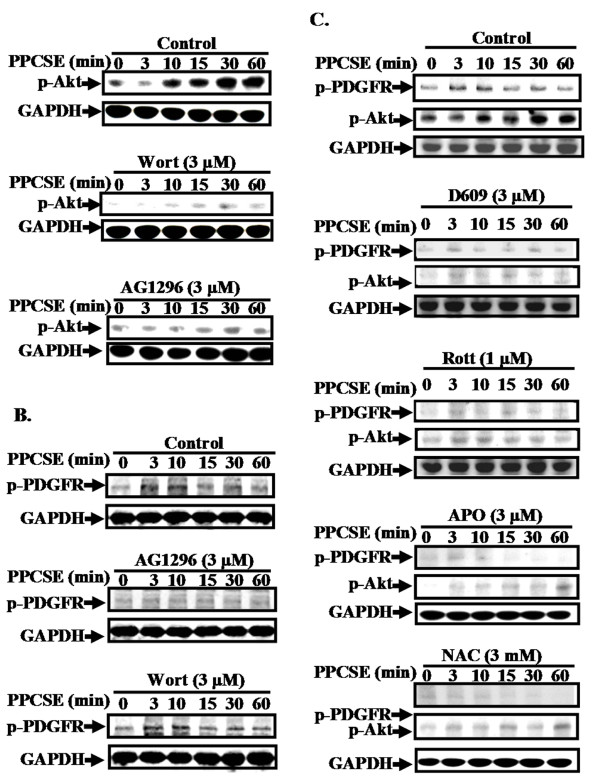
**PPCSE-induced HO-1 expression is mediated via a NADPH oxidase/ROS/PDGFR/PI3K/Akt signaling in bEnd.3 cells**. **A, B**: Cells were pretreated without or with wortmannin (Wort, 1 μM) or AG1296 (3 μM) for 1 h, and then stimulated with 30 μg/ml PPCSE for the indicated time intervals. The cell lysates were analyzed by Western blot using an anti-phospho-PDGFR, anti-phospho-Akt, or anti-GAPDH antibody. **C**: Cells were pretreated without or with D609 (an inhibitor of PC-PLC), rotterin (an inhibitor of PKCδ), APO (an inhibitor of NADPH oxidase), or NAC (a ROS scavenger) for 1 h, and then stimulated with 30 μg/ml PPCSE for the indicated time intervals. The phosphorylations of Akt and PDGFR were determined by Western blot. Similar results were obtained from three independent experiments.

PPCSE has been shown to stimulate phosphorylation of PDGFR and Akt leading to expression of several target proteins such as HO-1. To investigate if there was a cross-talk between PDGFR and Akt phosphorylation, wortmannin (inhibitor for PI3K) and AG1296 (inhibitor for PDGFR) were used. As shown in Figure 6A, PPCSE stimulated Akt phosphorylation in a time-dependent manner, which was attenuated by pretreatment with inhibitor of either PI3K (wortmannin) or PDGFR (AG1296) during the period of observation. However PPCSE-stimulated phosphorylation of PDGFR was not inhibited by wortmannin (Figure 6B). The data indicated that PI3K/Akt was a downstream component of PDGFR for PPCSE-induced HO-1 expression in bEnd.3 cells. We further clarified the involvement of PC-PLC/PKCδ/NADPH oxidase/ROS in PPCSE-stimulated activation of PDGFR/PI3K/Akt pathway. As shown in Figure 6C, pretreatment with either D609 (an inhibitor of PC-PLC), rottlerin (an inhibitor of PKCδ), APO (an inhibitor of NADPH oxidase) or NAC (a ROS scavenger) significantly reduced PPCSE-stimulated phosphorylation of PDGFR and Akt. These results suggested that PPCSE-stimulated PDGFR/PI3K/Akt cascade was mediated via PC-PLC/PKCδ/NADPH oxidase/ROS signaling pathway.

## Discussion

HO-1 upregulation has been observed in various types of cells exposed to diverse agents. Although PPCSE-induced oxidative stress and HO-1 expression has been extensively studied, the molecular mechanisms by which PPCSE induces oxidative stress and HO-1 expression are not fully understood in brain endothelial cells. In this study, we used murine brain endothelial cells (bEnd.3) as a model to demonstrate that the levels of HO-1 protein and their activity were increased upon exposure to PPCSE. In the present study, western blot analysis coupling with pharmacological inhibitors and transfection with dominant negative mutants revealed that PPCSE-induced HO-1 expression was regulated by activation of PDGFR-dependent PI3K/Akt pathway, which was mediated through PKCδ/NADPH oxidase/ROS. These results suggest that PKCδ/NADPH oxidase/ROS-dependent PDGFR/PI3K/Akt activation could play an important role in PPCSE-induced HO-1 expression in bEnd.3 cells.

Cerebral endothelial dysfunction induced by oxidative stress [29,30] or excitoneurotoxicity [31] has been shown to be diminished by HO-1 and also shown to be beneficial for cerebral ischemic injury [32,33]. However, HO-1 has been recently demonstrated to exacerbate early brain injury produced by intracerebral haemorrhagic stroke [6]. Hence, HO-1 may play different roles in ischemic or haemorrhagic stroke injury. PPCSE-induced HO-1 expression in cerebrovascular cells is therefore an important issue for brain injury in the field of CS toxicity.

It should be noted that the PPCSE used in this study was collected on a Cambridge filter, which traps particulate matter but not the gas phase. Within the particulate of PPCSE, lipophilic components are also present other than ROS. Lipophilic components are able to pass the lipid bilayer [1]. Therefore, it is possible that particulate matter of PPCSE dissolved in blood stream can reach brain vessels. Hence, the highly reactive lipophilic components such as polycyclic aromatic hydrocarbons, aldehydes, phenols, heavy metals, and amines could easily enter the brain through the blood-brain barrier.

Several isoforms of PKC have been found to mediate several cellular responses [34]. On the other hand, PKC has been also shown to regulate HO-1 expression [35]. Recently, PPCSE has been shown to activate PKC isoforms in rat bronchial smooth muscle cells [36]. In the present study, Ca^2+^-independent PKC, PKCδ, mediated HO-1 induction in response to PPCSE, which was confirmed by transfection of cells with a dominant negative mutant of PKCδ. PLC enzymes catalyze the cleavage of phospholipids and result in the release of diacylglycerol (DAG) in response to receptor activation and the DAG can trigger the activation of PKC [28]. In the present study, we found that D609 inhibited HO-1 expression induced by PPCSE, suggesting that PPCSE-induced HO-1 expression was mediated through PC-PLC. On the other hand, according to the kinetic studies, a maximal activation of PC-PLC was obtained within 1-3 min (Figure 4C), while a maximal activation of PKCδ was obtained within 3-10 min (Figure 3F). In addition, PKCδ translocation was attenuated by D609 (Figure 4D). These data suggested that PC-PLC may be an upstream component of PKC in these responses. Taken together, these results suggested that PPCSE-induced HO-1 expression was mediated through PC-PLC/PKCδ pathway in bEnd.3 cells.

The NADPH oxidase systems contribute as a major source of O_2_^.-^, which are present in endothelial cells, smooth muscle cells, and infiltrated monocytes/macrophages [37,38]. Oxidative stress occurs when the flux of ROS or free radical generation exceeds available antioxidant defenses. The NADPH oxidase family members are proteins that transfer electrons across biological membranes. In general, the electron acceptor is oxygen and the product of the electron transfer reaction is a superoxide. Therefore, the biological function of NADPH oxidase might be attributable to the production of ROS [39]. The aim of the present study was to examine whether the production of ROS in response to PPCSE that depends on NADPH oxidase activity led to the expression of HO-1. Previous study indicated that one of the early events occurring in smoke-exposed cells is the generation of ROS [40]. In the present study, ROS generation induced by PPCSE was attenuated by pretreatment with the inhibitors of NADPH oxidase, suggesting that PPCSE induced ROS production in an NADPH oxidase-dependent manner.

Moreover, it has been shown that PKC-dependent p47^phox ^phosphorylation and translocation are major mechanisms involved in NADPH oxidase activation [37] and ROS production [41,42]. Consistent with these studies, our results further showed that PPCSE-enhanced NADPH oxidase activity was attenuated by pretreatment with rottlerin, a PKCδ inhibitor. The involvement of PKCδ in PPCSE-mediated response was further confirmed by trasfection with dominant negative mutant of PKCδ which attenuated HO-1 expression. These results suggest that PPCSE-stimulated NADPH oxidase/ROS generation and HO-1 expression was mediated through activation of PKCδ.

It has been reported that several GPCRs initiate the PI3K/Akt pathway through transactivation of the EGFR or PDGFR in various cell types [43,44]. The downstream Akt phosphorylation was also mediated through transactivation of these growth factor receptors [45]. In the present study, PPCSE induced the phospharylation of Akt, with a maximal response within 30 min (Figure 6A). In addition, PPCSE stimulated the translocation of Akt from the cytosol to the nucleus, with a maximal response within 30 min (Figure 5E). On the other hand, a maximal phosphorylation of PDGFR was obtained within 3-10 min (Figure 6B). The phosphorylation of Akt induced by PPCSE was also inhibited by pretreatment with AG1296 (Figure 6A). These data suggested that PDGFR is an upstream component of Akt in PPCSE-mediated responses. CS can activate several signaling pathways including EGFR/PDGFR [46]. Our recent report with AG1296 and PDGFR shRNA demonstrated that PDGFR plays an important role in PPCSE-induced HO-1 expression in bEnd.3 cells [11]. However, little was known about the mechanisms of PPCSE that initiates the expression of HO-1 mediated through NADPH/ROS-dependent transactivation of PDGFR/PI3K/Akt in bEnd.3 cells. Our present results demonstrated that pretreatment with NAC or APO significantly reduced PPCSE-stimulated phosphorylation of PDGFR or Akt, suggesting the involvement of NADPH/ROS in PPCSE-induced PDGFR/PI3K/Akt activation. In addition, PKCδ was involved in PPCSE-induced NADPH oxidase activation, suggesting that PPCSE-induced HO-1 expression was mediated through a PKCδ/NADPH oxidase/ROS/PDGFR signaling. In addition to these signaling components involved in PPCSE-induced responses, MAPKs have been shown to regulate HO-1 expression in various cell types [47,48]. Thus, the relationship between PKCδ/NADPH oxidase/ROS and MAPKs in response to PPCSE will be further investigating in bEnd.3 cells.

It has been shown that a known toxin in tobacco smoke, acrolein, induced HO-1 expression through two independent pathways, PKCδ or PI3K, in human bronchial epithelial cells [18]. In that report, PKCδ appears to regulate HO-1 induction via modulating Nrf2 nuclear translocation, while PI3K may work through targeting on downstream signaling molecules other than Nrf2. However, in the present study, our results demonstrated that PKCδ activated PDGFR/PI3K/Akt signaling mediated through NADPH oxidase/ROS in bEnd.3 cells. The discrepancy may be due to cellular specificity or difference between pure compound and mixture.

## Conclusions

**Figure 7:**
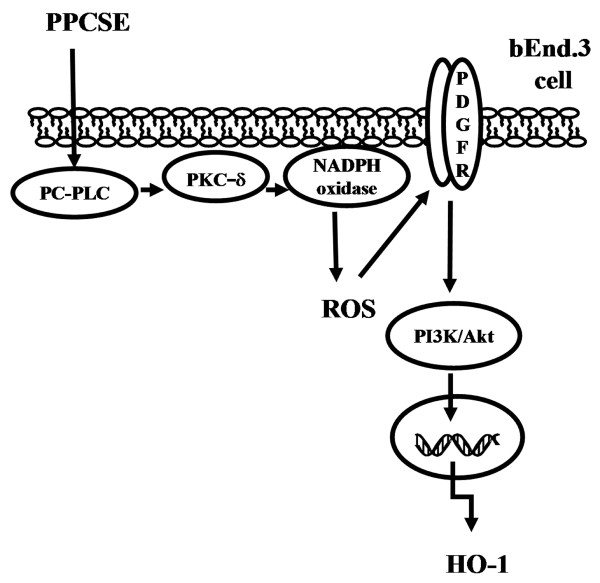
**PPCSE induces HO-1 expression through PDGFR/PI3K/Akt cascade in a PC-PLC/PKCδ/NADPH oxidase/ROS-dependent manner in bEnd.3 cells**. Schematic diagram illustrating the proposed signaling pathway involved in PPCSE-induced HO-1 expression in bEnd.3 cells. PPCSE-induced HO-1 expression is mediated by PC-PLC/PKCδ/NADPH oxidase/ROS-dependent PDGFR/PI3K/Akt cascade.

In summary, as depicted in Figure 7, our results showed that in bEnd.3 cells, PPCSE-induced HO-1 expression was, at least in part, mediated through PC-PLC/PKCδ/NADPH oxidase/ROS-dependent PDGFR/PI3K/Akt signaling pathway.

## Competing interests

The authors declare that they have no competing interests.

## Authors' contributions

RHS designed and performed experiments, acquisition and analysis of data, and drafted the manuscript. SEC and LDH helped to perform experiments and prepare the manuscript. YRK contributed to provide the particulate phase of cigarette smoke extract (PPCSE). CMY has conceived of the study, participated in its design and coordination, has been involved in drafting the manuscript and revising it critically for important intellectual content and have given final approval of the version to be published. The authors have read and approved the final version of this manuscript.
